# Evaluating the rate of reversal of fentanyl-induced respiratory depression using a novel long-acting naloxone nanoparticle, cNLX-NP

**DOI:** 10.3389/fpsyt.2024.1366186

**Published:** 2024-03-14

**Authors:** Saadyah E. Averick, Andrew J. Kassick, Daihyun Song, Borui Zhang, Jennifer Vigliaturo, Diego Luengas, Pedro Silva-Ortiz, Marco Pravetoni, Michael D. Raleigh

**Affiliations:** ^1^ Neuroscience Disruptive Research Lab, Allegheny Health Network Research Institute, Allegheny General Hospital, Pittsburgh, PA, United States; ^2^ Department of Pharmacology, University of Minnesota Medical School, Minneapolis, MN, United States; ^3^ Department of Psychiatry and Behavioral Sciences, University of Washington School of Medicine, Seattle, WA, United States; ^4^ Center for Medication Development for Substance Use Disorders, University of Washington, Seattle, WA, United States; ^5^ Garvey Institute for Brain Health Solutions, University of Washington, Seattle, WA, United States

**Keywords:** fentanyl, synthetic opioids, antagonist, reversal agent, antidote, substance use disorder, opioid use disorder

## Abstract

**Introduction:**

Fentanyl and fentanyl analogs (F/FA) have become increasingly common adulterants in counterfeit prescription pills and illicit street drug mixtures due to their ease of synthesis and exceedingly high potency. The ongoing epidemic of fatal overdoses fueled by F/FA continues to highlight the need for longer-acting therapies than naloxone (NLX), the current gold-standard for reversing opioid overdoses, which shows limited efficacy to prevent renarcotization associated with F/FA toxicity. A novel opioid reversal agent based on covalent naloxone nanoparticles (cNLX-NP) has been shown to blunt fentanyl-induced respiratory depression out to 48 hr, demonstrating its potential therapeutic utility. The purpose of this study was to characterize how rapidly cNLX-NP reverses fentanyl-induced respiratory effects as well as the duration of its protective effects.

**Methods:**

Sprague Dawley male rats (n=6/group) were tested on an oximeter for baseline percent arterial oxygen saturation (%SaO_2_) challenged with 0.1 mg/kg SC fentanyl and 15 min later given 10 mg/kg IM doses of NLX, nalmefene (NLMF), or cNLX-NP and continuously monitored via oximetry for 10 minutes. One week later the experiment was repeated using a 1:1 mixture of NLX:cNLX-NP as the reversal agent in the rats that previously received NLX alone.

**Results:**

While both NLX and NLMF rapidly reversed %SaO_2_ to baseline within 1 min, rats that received cNLX-NP did not return to >90% SaO_2_ levels until 9 min after administration. Similarly, heart and breath rates returned to baseline within 1 min of treatment with NLX and NLMF but did not return to baseline until 10 minutes after cNLX-NP administration. In contrast, NLX:cNLX-NP reversed all fentanyl-induced respiratory depressive effects within one minute.

**Discussion:**

While cNLX-NP alone may not sufficiently reverse F/FA overdose in a timely manner, mixing free NLX with cNLX-NP can provide a mechanism to both rapidly reverse fentanyl-related effects and maintain extended protection against synthetic opioid toxicity. These data support further development of cNLX-NP as a fast-acting and long-lasting antidote to treat F/FA-induced respiratory depression and overdose, and potentially prevent renarcotization in humans.

## Introduction

Opioid overdoses have accelerated in recent years, with over 107,000 reported fatalities in 2021 alone (1). Fentanyl and fentanyl analogs (F/FA) have been implicated in over 70,000 of those deaths (1), demonstrating the serious public health threat posed by this class of compounds (2–8). Fentanyl was detected in over 90% of fatal overdoses in Massachusetts between 2017 and 2019 (9). F/FA have appeared in non-opioid overdose deaths as well, with F/FA being detected in two-thirds of benzodiazepine deaths in 2020 (10), demonstrating that F/FA are a growing concern beyond opioid use. These trends have continued, or worsened, through 2023 (11). One of the challenges of treating F/FA overdoses is due to the different pharmacokinetic and pharmacodynamic (PK/PD) profiles of F/FA compared to current reversal agents.

F/FA are extremely hydrophobic mu opioid receptor (MOR) agonists that rapidly distribute to tissues including the blood-brain barrier (BBB) and are readily absorbed via multiple routes of administration (6, 12–15). The half-life of F/FA varies greatly between compounds and across individuals. Fentanyl has a half-life of 1 – 9 hours (16–18), remifentanil has a half-life of 8 – 48 min (16, 17, 19), alfentanil has a half-life of 0.42 – 1.6 hours (16, 18), and carfentanil has a half-life of 6 – 7 hours (16, 20). The current antidotes to treat F/FA overdose are small molecule MOR antagonists such as naloxone and nalmefene. Naloxone is rapidly eliminated and has a relatively short half-life of between 30 – 120 min (21–23). A recent study showed that naloxone occupancy of MORs decreased from 90% occupancy 5 min after administration to 50% occupancy 20 min later, which demonstrated how short-lived naloxone’s effects may be (24). Nalmefene, which is structurally similar to naloxone, has a much longer elimination half-life of approximately 11 hr in humans (25). This difference between the half-life of F/FA and naloxone could lead to renarcotization, whereby F/FA-induced respiratory depression returns after a brief period of reversal via treatment (20, 26, 27). This requires a subset of patients to be intubated and on ventilators in a hospital setting until they are capable of breathing on their own (20), or receive repeated doses of either naloxone or nalmefene. Because nalmefene was only recently FDA approved, it is not clear what role it will play in preventing renarcotization.

Other medications to treat F/FA overdose are at varying stages of preclinical development. Monoclonal antibodies (mAbs) against F/FA’s have demonstrated efficacy to reverse fentanyl and carfentanil effects (28, 29). Monoclonal antibodies provide the advantage of not blocking opioid receptors and a lesser likelihood of precipitating withdrawal. However, F/FA-specific mAbs would likely be more selective to single drug targets (instead of broadly reversing all opioid-induced effects) and may be less amenable to over-the-counter use due to logistics associated with administration routes and storage conditions. Methacinnamox (MCAM), another long-lasting opioid antagonist in preclinical development, has shown efficacy in reversing fentanyl-induced respiratory depression in rats and non-human primates (NHP) (30, 31) and had even longer efficacy against heroin-induced respiratory depression in NHP (32). However, MCAM may also precipitate withdrawal (33), and it has not yet been tested in humans. Further studies for these and other promising medications are warranted (34).

A new long-acting *covalently-linked* naloxone nanoparticle (cNLX-NP) has been developed as an antidote against F/FA, which demonstrated reversal of fentanyl-induced respiratory depression within 15 minutes of dosing and was protective against effects of fentanyl out to 48 hr after cNLX-NP administration (35). The cNLX-NPs are prepared via a two-step process involving the synthesis of biodegradable, naloxone-containing polymers and the subsequent precipitation of those polymers via a single-phase emulsion and nanoprecipitation to form well-defined nanoparticles. First, naloxone was ligated to the chain end of a biodegradable polymer via a hydrolytically labile bond, and then the polymer-bound NLX is then formulated as a nanoparticle using traditional fabrication methods wherein naloxone was non-covalently trapped in a particle (35). Given that polymer composition influences the biodegradation rate and controls the percent of naloxone incorporated in the nanoparticle, we synthesized a series of naloxone polymers possessing different levels of steric hinderance along the polymer backbone to control drug release. The steric properties were controlled by varying the amount of lactide (L) and glycolide (G) monomers that were grown from the 3-hydroxyl moiety of the naloxone initiator. Presented herein are two covalently loaded naloxone polymers with a 60:40 and 100:0 L:G composition.

This poly-lactic acid (PLA) and poly-lactic-co-glycolic acid (PLGA) nanoparticle technology is an advancement over current FDA-approved controlled-release drug delivery pharmaceuticals (such as Vivitrol^®^), which are in *non-covalently* drug-encapsulated form. Vivitrol^®^ contains PLGA particles loaded with naltrexone and displays an early burst release of antagonist followed by latent low release designed for treatment of opioid use disorder (OUD). If given post-exposure, this effect can lead to precipitated opioid withdrawal, as well as provide limited therapeutic coverage post injection. By contrast, the *covalent* nanoparticle formulation cNLX-NP provides a linear release of antagonist at a tunable rate for a long period of time providing consistent protection from renarcotization.

The goal of these studies was to determine the effect of altering polymer composition on naloxone half-life. The lead cNLX-NP formulation would be taken forward to compare against naloxone and nalmefene to elicit rapid reversal (within minutes) and long-lasting protection (prevention of renarcotization out to 48 hr) against fentanyl. Results demonstrated that the polymer containing only lactide and no glycolide provided naloxone the longest half-life, and that this formulation was protective out to 48 hr. However, cNLX-NP could not rapidly reverse fentanyl-induced respiratory depression, which was improved when free naloxone was added to the formulation. Together, these data suggest cNLX-NP may provide better protection than free naloxone or nalmefene to prevent F/FA-induced renarcotization.

## Materials and methods

### Ethics statement

All animal studies were performed in accordance with the Guide for the Care and Use of Laboratory Animals of the National Institutes of Health. Animal protocols were approved by the Allegheny General Hospital and University of Minnesota Institutional Animal Care and Use Committees. Animals were euthanized by CO_2_ inhalation using AAALAC approved chambers, and all efforts were made to minimize suffering.

### Formulation of cNLX-NP

Naloxone was used as an initiator for the ring opening polymerization of lactide or combinations of lactide and glycolide using previously reported conditions (35) (*Characterization of prepared polymers can be found in* **Supplementary Figures S1**, **S2**). Nanoparticles were formulated by slow injection of an acetonitrile solution of naloxone-containing polymer into a 0.3% polyvinyl alcohol solution (**Supplementary Figure S3**). Naloxone loading was calculated via hydrolysis of the particles and measurement of the released naloxone content (**Supplementary Table S1**). Particles were washed using water washing and centrifugation and freeze dried. Incorporation of glycolide increased the rate of nanoparticle hydrolysis.

### Nanoparticle preparation

**Figure 1:**
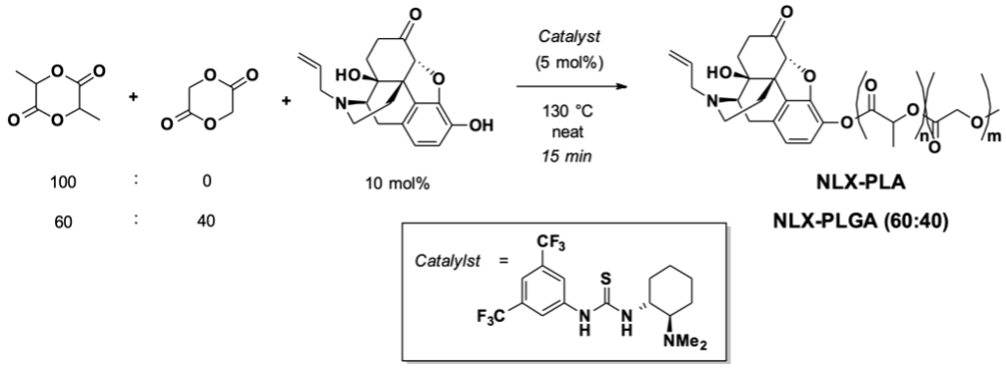
Synthesis of naloxone-PLA and naloxone-PLGA_60:40_ (cNLX-NP precursors) using ring opening polymerization.

Naloxone-containing polymers were synthesized according to the previously described solvent-free (35), organocatalyzed ring-opening polymerization (ROP) protocol as illustrated in **Figure 1**. Lactide and glycolide monomers were mixed at either 100:0 or 60:40 mole ratio, pre-melted at 130°C under an inert atmosphere, then treated with a mixture of naloxone (10 mol%) and thiourea catalyst (5 mol%). After 15 min, the reaction mixture was dissolved in CH2Cl2 and precipitated into cold isopropyl alcohol. The resulting precipitate was dried under reduced pressure and then subjected to flash chromatography. Characterization of polymers was achieved using gel permeation chromatography (GPC, **Supplementary Figure S1**) and proton nuclear magnetic resonance spectroscopy (^1^H NMR, **Supplementary Figure S2**) and the corresponding data is presented in **Table 1**. Isolated polymers were taken up in acetonitrile and added slowly dropwise via syringe pump to a 0.3% aqueous solution of poly(vinyl alcohol) (PVA_MW~6000_) with vigorous stirring. Dialysis against water with a 50 kDa MWCO membrane followed by lyophilization proved to be a suitable strategy to purify the nanoparticles. Particles were characterized using dynamic light scattering (**Supplementary Figure S3**, **Supplementary Table S2**) and hydrolyzed and naloxone concentration was measured using UV-Vis spectroscopy to determine naloxone loading percentage (**Supplementary Table S1**).

**Table 1:** GPC and ^1^H NMR characterization data for NLX-PLA and NLX-PLGA-based polymers (precursors of cNLX-NP).

Polymer	Yield (%)	*M_n_*	*M_w_*	*M_w_/M_n_*	DP	NLX loading (%)
NLX-PLA	53	4100	5200	1.26	36	11
NLX-PLGA (60:40)	30	5200	7500	1.44	64	7

### Drugs

Fentanyl citrate (Hikma Pharmaceuticals, previously West-Ward Pharmaceuticals, Berkeley Heights, NJ) and naloxone hydrochloride (LGM Pharma, Boca Raton, FL) were purchased from Boynton Pharmacy at the University of Minnesota. Naloxone hydrochloride dihydrate was purchased from LGM Pharma (LGM Pharma, Boca Raton, FL) and subsequently converted to the corresponding free base (2) via acid−base extraction with saturated aqueous sodium bicarbonate (NaHCO3). Nalmefene hydrochloride (Tocris, Minneapolis, MN) was purchased from Bio-Techne (Minneapolis, MN). Covalently loaded naloxone nanoparticles (cNLX-NP_60:40_ and cNLX-NP_100:0_, *written with a subscript reflecting the ratio of lactide:glycolide in their polymeric backbone*) were prepared as previously described (35).

### Animals

For Experiment 1, male Sprague−Dawley rats (300 g) were purchased from Charles River Laboratories (Wilmington, MA) with indwelling jugular vein catheters. For Experiments 2 and 3, male Sprague Dawley rats (8 weeks old) were purchased from Envigo (Indianapolis, IN). Rats were housed in AAALAC-approved facilities with a 14-hour light/10-hour dark cycle with free access to water and food.

### Analysis of drug levels

The concentrations of naloxone (from either free naloxone or cNLX-NP formulations), nalmefene, and fentanyl in the serum and brain from rats in Experiment 3 were measured by LC-MS/MS as described previously (35, 36). The limit of detection for naloxone and nalmefene was 1 ng/mL and the limit of quantitation for both was 2.5 ng/mL. Briefly, solid-phase extraction was used to isolate the drugs of interest from serum and brain using Bond Elut Plexa PCX cartridges (Agilent, Santa Clara, CA). The reconstituted samples were then analyzed by an Agilent G6470A triple quadrupole LC-MS/MS system consisting of an Infinity II 1290 G7116B Multicolumn Thermostat, G7120A High Speed Quad Pumps, and a G7267B Multisampler. Samples were stored at -20 °C until measurement.

### Respiratory depressive and antinociception assays

Fentanyl-induced respiratory depression and antinociception were measured by the pulse oximetry and hot plate test, respectively, as previously described (35). Briefly, after habituation to the testing environment, an oximetry collar (MouseOX, Starr Life Sciences Corp, Oakmont, PA) was placed around the neck of each rat to measure oxygen saturation (% SaO_2_) and heart rate at baseline and at various intervals based on experimental details (see specific experimental details below). Immediately following each oximetry measurement, rats were placed on a hot plate (Columbus Instruments, Columbus, OH) set to 54°C to measure latency to respond (as indicated by a lift or flick of the hind paw or jumping) with a maximum cutoff of 30 sec to avoid thermal tissue damage.

#### Experiment 1 - Pharmacokinetics of naloxone nanoparticles compared to current therapeutics

To determine the elimination half-life of nalmefene and cNLX-NP_100:0_, male Sprague-Dawley rats (n=3-6) were given a normalized dose of 10 mg/kg reversal agent (nalmefene or cNLX-NP_100:0_) via intramuscular (IM) administration. Blood was drawn at t = 0, 0.5, 1, 1.5, 2, 3, 4, 6, 9, 12, 24, 48, and 72 hr at a volume of 0.1 mL per time point. Catheters were flushed with 0.2 mL of heparin (50 IU/ml) and locked to maintain catheter patency. Naloxone and cNLX-NP_60:40_ pharmacokinetic parameters were previously published (35) but were included in the present study because the experiment was identical and new analyses were performed. Any place where previously published data are presented will be noted.

#### Experiment 2 - Long-term efficacy of opioid antagonist reversal agents

To determine the long-term efficacy of cNLX-NP_100:0_ to reverse fentanyl-induced respiratory depression, rats (n=6/group) were baselined on a hotplate set to 54°C and monitored by oximetry (MouseOX) for oxygen saturation (% SaO_2_ and heart rate) prior to experiment on each day. Then, 0.1 mg/kg SC fentanyl was given at t=0, 6, 24, and 48 hr. Fifteen minutes after fentanyl administration, rats were monitored on the hotplate for antinociception and via oximetry. Immediately afterwards at t=17 min (but not at t=6, 24, or 48 h), rats received a single IM dose of 10 mg/kg naloxone, nalmefene, or cNLX-NP_100:0_. On Day 1 (t=30 min), rats were monitored on the hotplate and oximeter to demonstrate that all formulations rapidly reversed fentanyl effects. On Day 2 (t=24 h), rats were tested on the hotplate and oximeter 15 minutes after fentanyl exposure and any rats that had <90% SaO_2_ were given a 0.1 mg/kg SC dose of naloxone to reverse fentanyl-induced respiratory depression. On Day 3 (t=48hr), rats were tested on the hotplate and oximeter 15 minutes after fentanyl exposure and immediately euthanized to measure fentanyl, naloxone, and nalmefene levels in serum and brain via LCMS. See **Supplementary Figure S4** for the study design.

#### Experiment 3 - Rapid reversal of fentanyl-induced effects using opioid antagonists.

To determine the rate of reversal of cNLX-NP formulations, rats (n=6/group) were baselined via oximetry and then given 0.1 mg/kg fentanyl SC. Fifteen minutes later rats were tested again on the oximeter. Immediately afterwards, rats received 10 mg/kg IM of naloxone, nalmefene, or cNLX-NP_100:0_. One week later, this experiment was repeated in six rats (two rats from each group that were randomly selected) who received a 1:1 mixture of 5 mg/kg free naloxone and 5 mg/kg cNLX-NP_100:0_.

### Statistics

Pharmacokinetic parameters in Experiment 1 were estimated using noncompartmental analysis using PKSolver, an excel-based pharmacokinetic software as previously described (37, 38), and using Phoenix WinNonlin (Certara, Princeton, NJ), the benchmark software application for pharmacokinetics. Within each analysis type (PKSolver or WinNonlin) groups were compared using one-way ANOVA with Welch’s correction. To compare between analysis types (PKSolver versus WinNonlin), multiple t test analysis was performed. Oxygen saturation, heart rate, and antinociception were compared between groups at each time point in Experiment 2 using two-way ANOVA using Tukey’s multiple comparison test. Naloxone and nalmefene levels were compared between groups in serum or brain in Experiment 2 using the Kruskal-Wallis one-way ANOVA.

## Results

### Experiment 1: Pharmacokinetics of naloxone nanoparticles compared to current therapeutics

**Figure 2:**
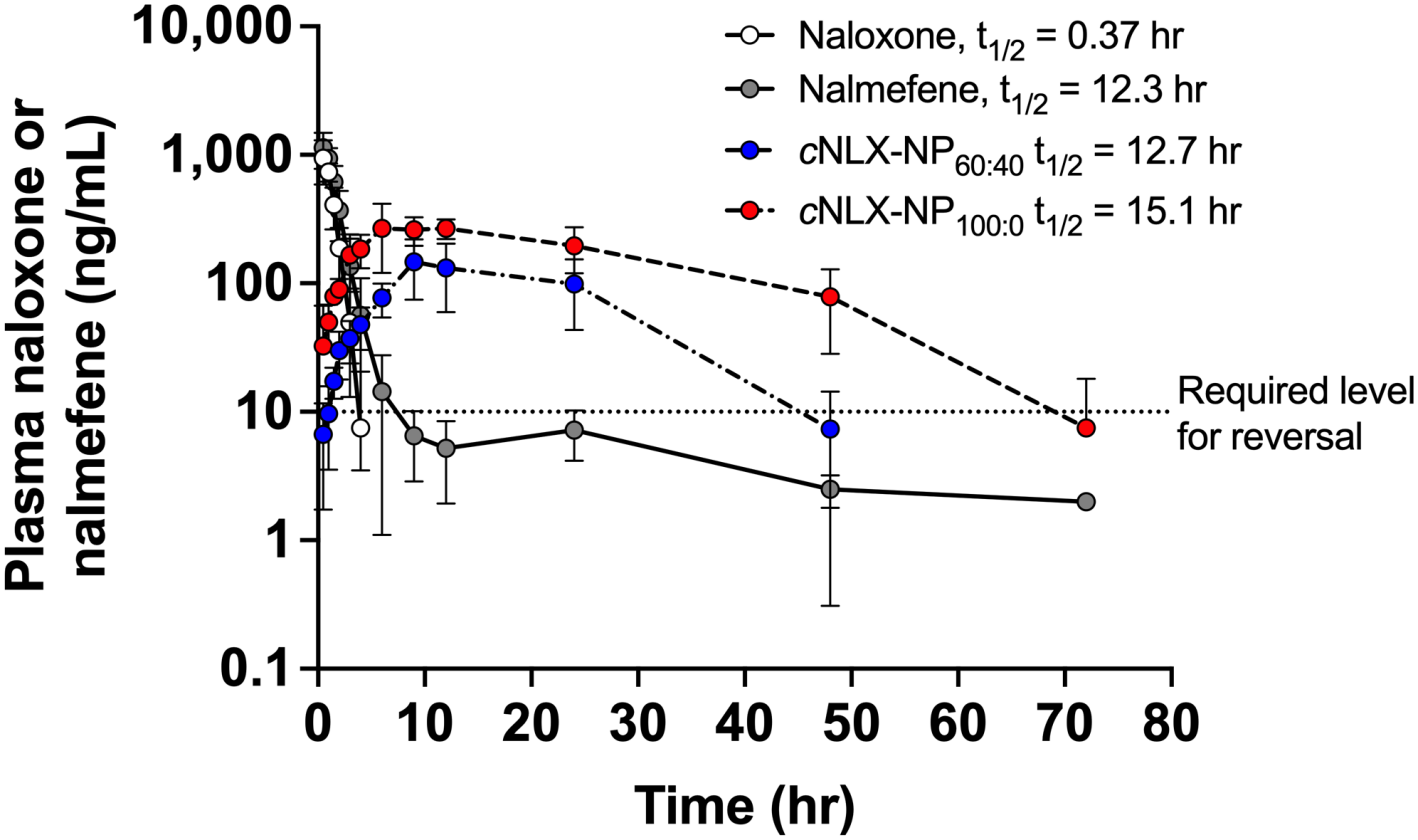
Pharmacokinetics of opioid antagonist reversal agents. Rats (n=2-6/group at each time-point) were given IM doses of 10 mg/kg of naloxone, nalmefene, cNLX-NP_60:40_, or cNLX-NP_100:0_ and blood was taken at various time points as shown. Naloxone and cNLX-NP_60:40_ data were previously published (35) and were shown for comparison. Half-life was calculated using PKSolver. The dotted line on the Y-axis at 10 ng/mL estimates the amount of naloxone or nalmefene needed to significantly reduce fentanyl-induced respiratory depression in rats (as demonstrated in Experiment 2). Data are represented as Mean ± SD.

The pharmacokinetic parameters of opioid antagonists were explored. After administration of a 10 mg/kg dose, naloxone showed a rapid reduction in drug plasma levels with a terminal half-life of 0.37 ± 0.05 hr. Nalmefene, cNLX-NP_60:40_, and cNLX-NP_100:0_ exhibited much longer half-lives relative to naloxone at 12.3 ± 11.1 hr (33-fold increase), 12.67 ± 5.38 hr (34-fold increase), and 16.90 ± 2.54 hr (46-fold increase), respectively (**Figure 2**). However, only cNLX-NP_100:0_ showed a statistical difference compared to naloxone (F_(3,7.9)_=4.86, p<0.05). Other pharmacokinetic parameter comparisons are shown in **Table 2**. Because cNLX-NP_100:0_ had the best pharmacokinetic profile, it was selected as the lead formulation in subsequent experiments.

**Table 2:** Comparison between PKSolver and WinNonlin pharmacokinetic parameters of opioid antagonist reversal agents from Experiment 1.

	Parameter	Naloxone^2^ (n=3)	Nalmefene (n=6)	cNLX-NP_60:40_^2^ (n=3)	cNLX-NP_60:40_^3^ (n=2)	cNLX-NP_100:0_ (n=5)
**PKSolver**	Terminal t_1/2_ (*hr*)	0.37 ± 0.05	12.3 ± 11.1	12.67 ± 5.38	9.56 ± 0.02	16.90 ± 2.54*
	T_max_ (*hr*)	0.5 - 1.0	0.5	9	9	1 - 12
	C_max_ (*ng/mL*)	1010 ± 250.85	1132 ± 355	147 ± 72.5^+^	147 ± 72.5	377 ± 102.91^+ #^
	AUC 0-inf^1^ (ug/mL*hr)	1.25 ± 0.17	2.1 ± 0.61	3.97 ± 1.69	4.68 ± 1.62	10.73 ± 1.86* ^+ #^
	MRT 0-inf^1^ (*hr*)	1.11 ± 0.21	4.64 ± 2.75	23.51 ± 7.20	19.36 ± 0.37	24.11 ± 5.08* ^+^
	Cl/F^1^ (*ml/kg/min*)	135.05 ± 17.13	85.5 ± 22.8*	47.17 ± 18.65*	37.81 ± 13.07	15.87 ± 2.45* ^+^
**WinNonlin**	Terminal t_1/2_ (*hr*)	0.37 ± 0.05	12.4 ± 11	N/A	8.85 ± 0.10*	16.5 ± 2.82* ^#^
	T_max_ (*hr*)	0.5 - 1.0	0.5	N/A	9	1 - 12
	C_max_ (*ng/mL*)	1010 ± 251	1130 ± 355	N/A	147 ± 72.5^+^	378 ± 103^+^
	AUC 0-inf^1^ (ug/mL*hr)	1.2 ± 0.17	2.1 ± 0.6	N/A	4.16 ± 1.44	10.0 ± 2.1* ^+^
	MRT 0-inf^1^ (*hr*)	1.13 ± 0.2	4.80 ± 2.84	N/A	20.1 ± 0.48* ^+^	26.6 ± 3.40* ^+^
	Cl/F^1^ (*ml/kg/min*)	140.33 ± 18.17	87.8 ± 23.3	N/A	42.67 ± 14.72*	17.2 ± 3.02* ^+^

**^1^** Observed, ^2^PKSolver data previously published (35), ^3^Because one animal could not be analyzed with WinNonlin, the same animal was excluded from PKSolver and shown here for comparison between analysis types only. This group was not used to compare within the PKSolver analysis groups. Data are Mean ± SD, except T_max_, which is expressed as median. *p<0.05 compared to Naloxone; ^+^p<0.05 compared to Nalmefene; ^#^p<0.05 compared to cNLX-NP_60:40_ within analysis methods. No differences between PKSolver and WinNonlin analyses were observed. N/A, Not Available.

To ensure that the excel-based pharmacokinetic software (PKSolver) calculated the appropriate pharmacokinetic parameters of the reversal agents, data were compared against Pharsight WinNonlin (**Table 2**). No differences between the two packages were detected, although PKSolver was able to compute the pharmacokinetic parameters of one animal in the cNLX-NP_60:40_ group that WinNonlin could not. It was not clear why this difference occurred. By removing the animal from PKsolver, the two programs showed identical pharmacokinetic parameters in the cNLX-NP_60:40_ formulation.

### Experiment 2: Long-term efficacy of opioid antagonist reversal agents

**Figure 3:**
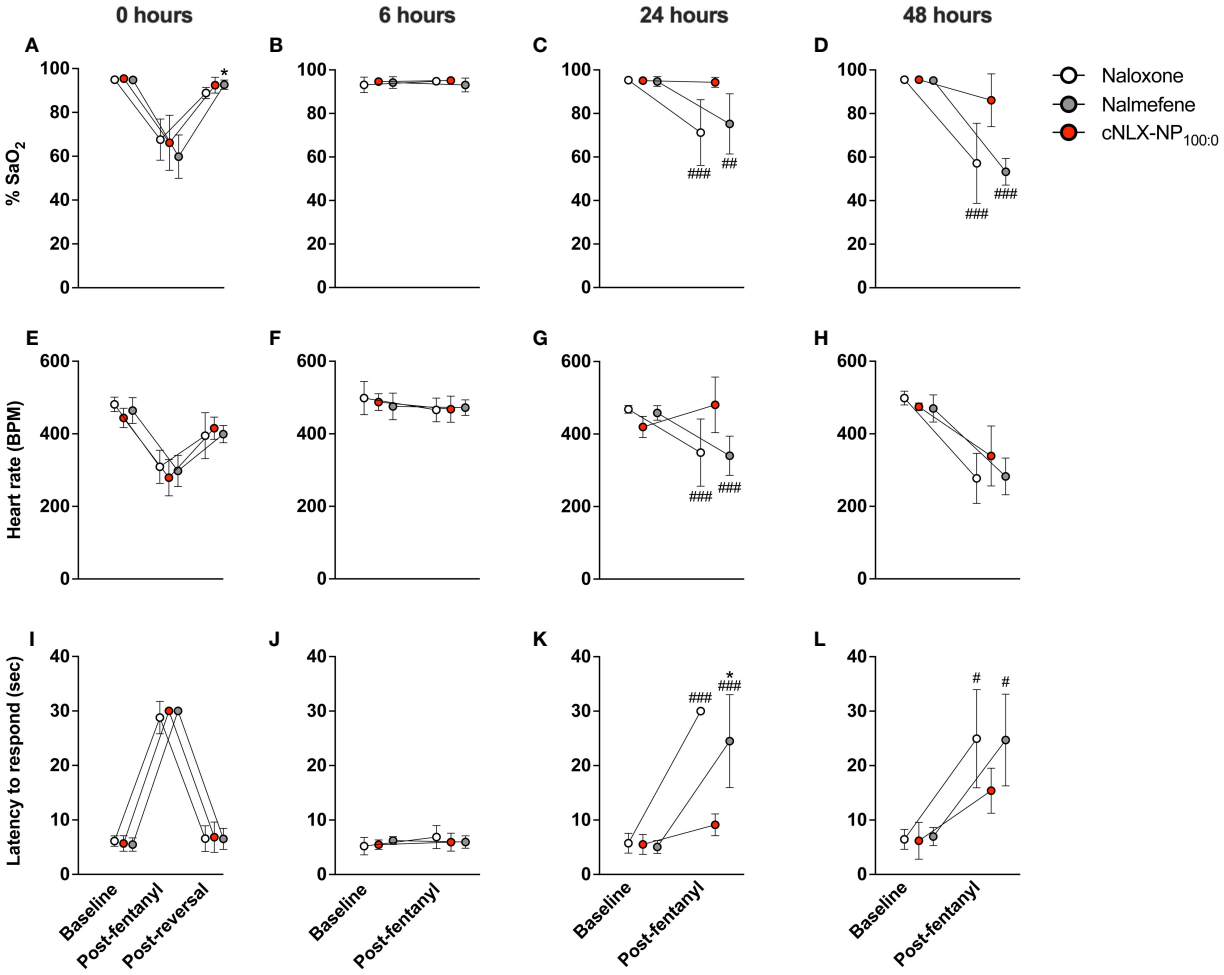
Long-lasting efficacy of cNLX-NP_100:0_ to reverse fentanyl-induced effects. Rats (n=6/group) received 0.1 mg/kg SC fentanyl at t=0, 6, 24, and 48 hr and 10 mg/kg IM naloxone, nalmefene, or cNLX-NP_100:0_ at t=17 min only and were monitored for physiological parameters via oximetry for %SaO_2_ **(A-D)** and heart rate **(E-H)** and for antinociception via hotplate using latency to respond to lick hindpaw or jump **(I-L)**. Results demonstrated that all formulations effectively reversed fentanyl-induced effects 15 minutes after administration (left-most panels) at t=0 hr and all formulations remained effective at preventing fentanyl-induced effects at t=6 hr (second column of panels from the left). Only cNLX-NP_100:0_ remained effective at preventing fentanyl-induced effects at t=24 and t=48 hr compared to naloxone and nalmefene. At t=24 hr, all animals that had previously received naloxone or nalmefene had oxygen saturation levels <90% post-fentanyl administration and required an additional SC dose of naloxone to reverse fentanyl-induced effects. Only one animal in the cNLX-NP_100:0_ group required naloxone. Data are represented as Mean ± SD.*p<0.05 compared to naloxone; ^#^p<0.05, ^##^p<0.01, and ^###^p<0.001 compared to cNLX-NP_100:0_ using two-way ANOVA.

**Figure 4:**
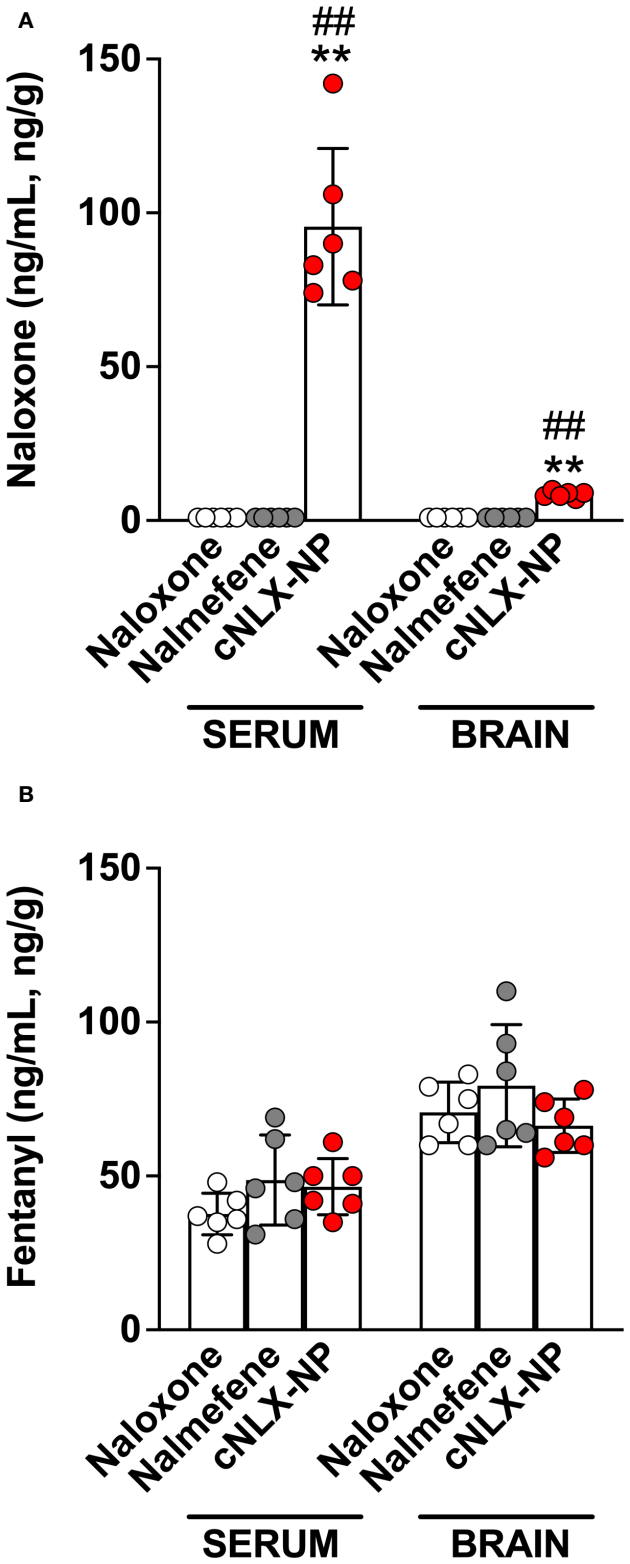
Opioid levels from Experiment 2 in serum and brain of rats 48 hr after administration. Following the t=48 hr fentanyl dose and hotplate and oximetry assessment, serum and brain were collected from all rats from *Experiment 2*. Naloxone and nalmefene serum and brain levels were undetected in all groups excect cNLX-NP, which showed high levels of free naloxone in serum 48 hr after administration **(A)**. No differences in fentanyl levels were detected in any groups treated with naloxone, nalmefene, or cNLX-NP **(B)**, confirming that effects demonstrated by cNLX-NP_100:0_ from **Figure 3** are due to the presence of free naloxone and not due to differences in fentanyl distribution. Data are represented as Mean ± SD. **p<0.01 compared to naloxone; ^##^p<0.01 compared to nalmefene in serum or brain using the Kruskal-Wallis multiple comparison ANOVA.

This study assessed the long-term protective effects of each antagonist to prevent fentanyl-induced respiratory depression after repeated exposures. At t=0 hr, naloxone, nalmefene, and cNLX-NP_100:0_ all reversed fentanyl-induced effects such as oxygen saturation (**Figure 3A**), bradycardia (**Figure 3E**), and antinociception (**Figure 3I**) 15 min after administration. Two-way ANOVAs for oxygen saturation, bradycardia, and antinociception only showed a significant effect on time (F_(1.1,16.6)_=124.6, p<0.0001; F_(1.8,26.5)_=77.7, p<0.0001; F_(1.8,26.7)_, p<0.0001, respectively). At t=6 hr, all reversal agents continued to completely prevent fentanyl effects (**Figures 3B, F, J**), with no significant differences between groups detected. At 24 hr, only cNLX-NP_100:0_ was effective at preventing fentanyl-induced respiratory depression, bradycardia, and antinociception (**Figures 3C, G, K**, respectively). Two-way ANOVA for oxygen saturation showed a significant effect on time (F_(1,15)_=32.0, p<0.0001), treatment (F_(2,15)_=5.5, p<0.05), and interaction (F_(2,15)_=7.4, p<0.01). Two-way ANOVA for bradycardia showed a significant effect on time (F_(1,15)_=10.2, p<0.01) and interaction (F_(2,15)_=10.6, p<0.01). Two-way ANOVA for antinociception showed a significant effect on time (F_(1,15)_=190.3, p<0.0001), treatment (F_(2,15)_=21.4, p<0.0001), and interaction (F_(2,15)_=29.8, 0.0001). Rats that had oxygen saturation levels <90% following the end of study at 24 hr required a dose of naloxone to prevent hypoxia. All rats from the naloxone and nalmefene groups (100%) required a dose of naloxone, while only 1 rat from the cNLX-NP_100:0_ group (17%) needed a dose. At 48 hr, only cNLX-NP_100:0_ was effective at preventing fentanyl-induced respiratory depression and antinociception (**Figures 3D, H, L**). Two-way ANOVA for oxygen saturation showed a significant effect on time (F_(1,15)_=90.8, p<0.0001), treatment (F_(2,15)_=11.3, p<0.001), and interaction (F_(2,15)_=10.7, p<0.01). Two-way ANOVA for bradycardia showed a significant effect on time (F_(1,15)_=99.7, p<0.001). Two-way ANOVA for antinociception showed a significant effect on time (F_(1,15)_=58.9, p<0.0001). Following the end of the study at 48 hr, fentanyl, free naloxone, and nalmefene levels were measured to demonstrate that no differences between fentanyl levels were observed between groups and to analyze antagonist levels. There was significantly more (p<0.01) free naloxone in the cNLX-NP_100:0_ group compared to both naloxone or the nalmefene groups 48 hr after administration (**Figure 4A**). Nalmefene levels in serum and brain were below the limit of detection for all subjects. Fentanyl levels were not different between groups in serum and brain (**Figure 4B**).

### Experiment 3: Rapid reversal of fentanyl-induced effects using opioid antagonists

**Figure 5:**
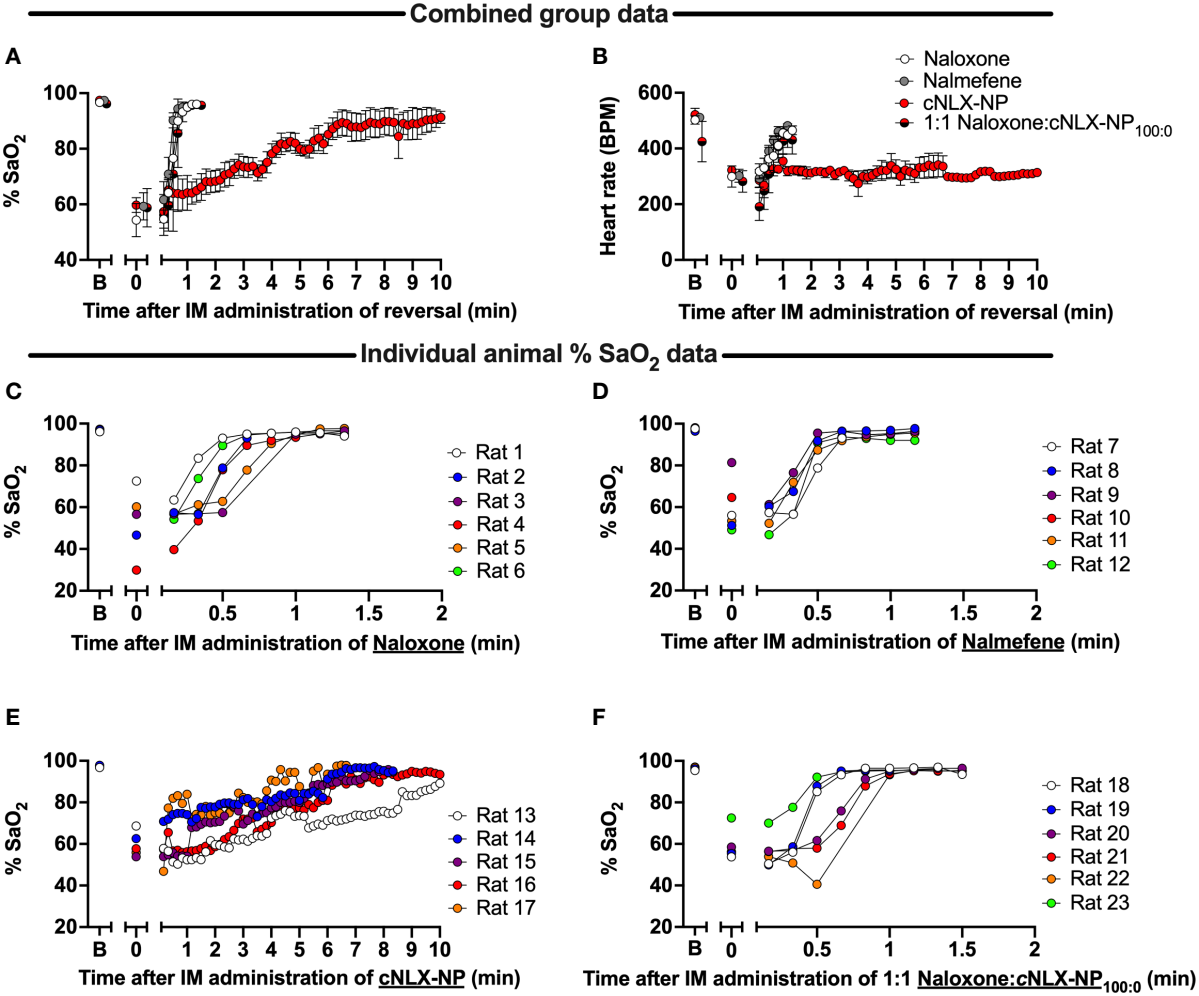
Time-course of reversal of fentanyl-induced respiratory depression and bradycardia following antagonist administration in rats. Rats (n=6/group) were baselined (x-axis label ‘B’) using a collar-based arterial pulse oximeter to obtain oxygen saturation levels (%SaO_2_) and then given 0.1 mg/kg fentanyl SC. Fifteen minutes later rats were tested again on the oximeter (x-axis label ‘0’). Immediately afterwards, rats received 10 mg/kg IM of naloxone, nalmefene, or cNLX-NP_100:0_ and % SaO_2_ **(A)** and heart rate measured **(B)**, represented as Mean ± SD. Results demonstrated that naloxone **(C)**, nalmefene **(D)**, but not cNLX-NP **(E)**, rapidly reversed fentanyl-induced respiratory depression. To improve recovery rate for cNLX-NP_100:0_, two rats (n=6) from each group were randomly selected and a week later the experiment was repeated using 5 mg/kg IM naloxone with 5 mg/kg IM cNLX-NP_100:0_ as the reversal agent. These data demonstrated that a 1:1 Naloxone and cNLX-NP_100:0_ formulation rapidly reversed fentanyl-induced respiratory depression **(F)** and that cNLX-NP_100:0_ does not interfere with efficacy of free naloxone.

Oxygen saturation (**Figure 5A**) and heart rate (**Figure 5B**) were monitored continuously post-reversal administration (10 mg/kg normalized concentration, IM) fifteen minutes after a SC fentanyl (0.1 mg/kg) challenge. Oxygen saturation levels rapidly returned to baseline levels within 1 min following naloxone (**Figure 5C**) and nalmefene (**Figure 5D**) administration, while the cNLX-NP formulation required at least 9 min to observe oxygen saturation levels of >90% or higher (**Figure 5E**). To determine if mixing free naloxone with cNLX-NP_100:0_ could improve antagonist efficacy, a 1:1 ratio of 5 mg/kg naloxone with 5 mg/kg cNLX-NP_100:0_ (for a total 10 mg/kg normalized naloxone) was formulated. This 1:1 mixture exhibited an identical recovery from fentanyl-induced respiratory depression as free naloxone (**Figures 5A, F**). Heart rate in the cNLX-NP_100:0_ group did not return to baseline levels within the measured time frame (**Figure 5B**).

## Discussion

The key findings of this study were that cNLX-NP_100:0_: 1) extended the terminal half-life of naloxone beyond that of naloxone alone or nalmefene; 2) blocked fentanyl effects out to 48 hr; and 3) was effective in rapidly reversing fentanyl-induced respiratory depression only when combined 1:1 with free naloxone. These data suggest that cNLX-NP_100:0_ may be a long-lasting therapeutic to prevent F/FA-induced renarcotization and, in combination with free naloxone, may be an effective formulation to rapidly reverse opioid-induced respiratory depression.

The pharmacokinetics of naloxone was previously published (35) and that data were presented here alongside a new formulation of cNLX-NP_100:0_ and nalmefene. The half-life of naloxone was measured at 22 min, which is well within the 15 – 30 min range in rats reported in the literature (23, 39). The half-life of naloxone was also reported following a 0.4 mg dose in humans at 64 min (23), suggesting naloxone has a slightly longer half-life in humans compared to rats. These differences likely do not impact the interpretation of the findings in the current study.

The current study also compared the pharmacokinetics of nalmefene, which was recently approved by the FDA for treatment of opioid overdose (40). In rats, the terminal half-life of nalmefene after a 5 mg/kg IV bolus dose was measured as 45 min (41). However, drug levels in blood were only measured out to 180 min, which may have artificially lowered the apparent half-life if only the alpha phase (which relates more to the redistribution of drug rather than the elimination) was measured. Although the current study measured the half-life of nalmefene after an IM dose, the half-life was 12.3 hours, which is 16X longer than the previous report in rats. It was apparent from **Figure 2** that nalmefene has a rapid redistribution phase but very slow elimination, which may not have been accounted for in the previous report. One study measured nalmefene half-life at 26 – 35 min in rats, but this was after nasal administration and was measured only out to 30 min following small doses between 15 – 45 μg/mL (42). No other reports on nalmefene pharmacokinetics in rats could be found. In humans, the terminal half-life of nalmefene around 8 – 11 hr following IV bolus doses between 2 – 24 mg (43, 44), which matches the rat data from the current study. These data suggest that nalmefene pharmacokinetics may be similar between humans and rats and that the current findings related to nalmefene and cNLX-NP could be extrapolated to humans.

An effective antidote to treat F/FA overdose and prevent renarcotization needs to have a half-life that is greatly superior to that of naloxone. In a previous publication describing the use of covalently loaded naloxone nanoparticles derived from PLGA (cNLX-NP_60:40_), it was shown that the half-life of naloxone could be extended by approximately 34-fold (35). The current study demonstrated that by using only *rac*-lactide instead of a mixture of *rac*-lactide and glycolide the half-life of naloxone could be extended about 33% more. Furthermore, cNLX-NP_100:0_ had approximately 10X more free naloxone at 48 hr than cNLX-NP_60:40_, suggesting that the *rac-*lactide formulation could perform even beyond 48 hr. These data are in accordance with the results of similar studies examining the biodegradation of various PLA and PLGA polymers. It has been well documented in the literature that PLA exhibits slow rates of hydrolysis while the hydrolysis of PLGA copolymers is considerably faster (45). In fact, increasing the glycolic acid content is a common strategy to increase the degradation rate of a polymer (46). This phenomenon can be best explained using a simple steric argument. The methyl-bearing stereocenter of lactic acid provides a more sterically crowded environment around the ester carbonyls in PLA thus hindering nucleophilic attack and subsequent hydrolysis (47). In the present study, this translates to a slower, sustained release of naloxone and a longer circulatory half-life. In contrast, glycolic acid ester groups do not possess any substitution on the adjacent α-carbon leaving them more readily accessible to nucleophilic attack and more prone to hydrolysis resulting in a shorter half-life as observed for cNLX-NP_60:40_. However, because the two naloxone nanoparticles (cNLX-NP_100:0_ and cNLX-NP_60:40_) were studied at separate times in different cohort of rats, although under the same conditions, caution should be applied when interpreting the results.

Although nalmefene and the cNLX-NP_100:0_ have similar half-lives, nalmefene was only effective out to 6 hr, while cNLX-NP_100:0_ reduced fentanyl-induced effects out to 48 hr. The cNLX-NP_60:40_ formulation previously performed similarly (35). Based on these results, a hypothetical effective threshold of antagonist needed to prevent fentanyl-induced respiratory depression of 10 ng/mL was added to **Figure 2** y-axis. Confirming that fentanyl levels were identical between groups at 48 hr, and that naloxone levels were only detected in the cNLX-NP_100:0_ group, demonstrated that efficacy of cNLX-NP_100:0_ was related to sustained release of naloxone and not due to any apparent differences in fentanyl levels. Although fentanyl has been observed to induce tolerance (35), post-fentanyl oxygen saturation data in the naloxone and nalmefene groups at t=0 hr and t=48 hr were similar and suggest that tolerance did not play a major role in the findings of this study. Combined, these data suggest that a long-acting nanoparticle-based formulation can outperform both naloxone and nalmefene, continuing to protect against F/FA-induced respiratory depressive effects beyond 6 hr.

PKSolver was demonstrated to produce pharmacokinetic parameters that were identical to the industry standard software package Phoenix WinNonlin, validating the use of this software package in the current study as well as previous studies (35, 38, 48). The original developers also validated this software against WinNonlin and showed that no differences were apparent (37). PKSolver is an add-on to Microsoft Excel which has an easier-to-use interface than WinNonlin. Further, Microsoft Excel is a software package readily available to most researchers whereas WinNonlin has expensive licensing restrictions that make it difficult to access. The downside to PKSolver is that the software package is no longer maintained, cannot be used in versions of Microsoft Excel beyond 2010, is not available on the Mac OS platform, and may require the use of virtualization within newer operating systems.

An important feature of an opioid antidote is the ability to rapidly reverse (i.e., within 1 min) an overdose. Although cNLX-NP_100:0_ was able to reverse fentanyl-induced respiratory depression 15 minutes after administration as measured in the current study, it was unable to rapidly reverse fentanyl effects within minutes to the same degree as naloxone and nalmefene. This was likely because no free naloxone was available immediately after administration. However, a mixture of 1:1 free naloxone with cNLX-NP_100:0_ demonstrated rapid reversal of fentanyl-induced respiratory depression, indicating that combining free antagonist with a nanoparticle formulation can provide immediate and long-lasting effects, while preserving the benefits of both therapeutics.

Bradycardia was rapidly reversed in all groups in Experiment 3, except the cNLX-NP_100:0_ alone group which failed to return to baseline levels within 10 minutes assessment period. This is surprising given that bradycardia was reversed within 15 minutes in Experiment 2. A potential difference between these two experiments is that rats were handled immediately prior to oximetry testing in Experiment 2, which may have artificially increased heart rate levels, while rats in Experiment 3 were untouched during measurement. Further testing may be needed to better understand the discrepancies between these experiments.

In this report, the pharmacokinetics and immediate and long-lasting efficacy of cNLX-NP formulations were compared against current opioid antagonists, naloxone and nalmefene. The results demonstrated that a formulation of cNLX-NP that contained only *rac-*lactide (cNLX-NP_100:0_) showed a better pharmacokinetic profile than a formulation containing both *rac-*lactide and glycolide (cNLX-NP_60:40_), suggesting that changes to the nanoparticle formulation can significantly alter the extent of free naloxone distribution. Mixture of free naloxone and cNLX-NP_100:0_ could rapidly reverse fentanyl-induced respiratory depression and cNLX-NP_100:0_ was effective out to 48 hr. Together, the data support cNLX-NP_100:0_ as a lead candidate opioid antagonist formulation to treat overdose and prevent renarcotization following exposure to fentanyl and fentanyl analogs in humans. Finally, this technology could be used to develop other chemical countermeasures for treating exposure to other chemical threats, such as toxins, nerve agents, or other chemical agents.

## Data availability statement

The original contributions presented in the study are included in the article/**Supplementary Material**. Further inquiries can be directed to the corresponding author.

## Ethics statement

The animal study was approved by Allegheny General Hospital and University of Minnesota Institutional Animal Care and Use Committees. The study was conducted in accordance with the local legislation and institutional requirements.

## Author contributions

SA: Conceptualization, Formal analysis, Funding acquisition, Investigation, Methodology, Project administration, Resources, Validation, Visualization, Writing – original draft, Writing – review & editing. AK: Formal analysis, Investigation, Methodology, Resources, Visualization, Writing – original draft, Writing – review & editing. DS: Investigation, Methodology, Writing – review & editing. BZ: Investigation, Writing – review & editing. JV: Investigation, Methodology, Validation, Writing – review & editing. DL: Investigation, Writing – review & editing. PS: Investigation, Writing – review & editing. MP: Conceptualization, Funding acquisition, Methodology, Writing – original draft, Writing – review & editing. MR: Conceptualization, Formal analysis, Funding acquisition, Investigation, Methodology, Project administration, Resources, Validation, Writing – original draft, Writing – review & editing.
