# Supplementary material for: Evaluating the rate of reversal of fentanyl-induced respiratory depression using a novel long-acting naloxone nanoparticle, cNLX-NP
Source: Front Psychiatry. 2024 Mar 14;15:1366186. doi: 10.3389/fpsyt.2024.1366186 (PMC10973107; doi:10.3389/fpsyt.2024.1366186)
Supplement: Supplementary file 1 [file DataSheet_1.docx]

**Evaluating the rate of reversal of fentanyl-induced respiratory depression using a novel long-acting naloxone nanoparticle, cNLX-NP.**

**Supplemental Information**

Saadyah E. Averick^1^, Andrew J. Kassick^1^, Daihyun Song^2^, Borui Zhang^1^, Jennifer Vigliaturo^2^, Diego Luengas^2^, Pedro Silva^2^, Marco Pravetoni^3,4,5^, and Michael D. Raleigh^2^*

^1^Neuroscience Disruptive Research Lab, Allegheny Health Network Research Institute, Allegheny General Hospital, Pittsburgh, PA, USA

^2^Department of Pharmacology, University of Minnesota Medical School, Minneapolis, MN, USA

^3^Department of Psychiatry and Behavioral Sciences, University of Washington School of Medicine, Seattle, WA, USA

^4^Center for Medication Development for Substance Use Disorders, University of Washington, Seattle, WA, USA

^5^Garvey Institute for Brain Solutions, Seattle, WA, USA

^#^Corresponding author: Michael Raleigh, [rale0011@umn.edu](mailto:rale0011@umn.edu). University of Minnesota, Department of Pharmacology, 3-121 Nils Hasselmo Hall, 312 Church St. SE, Minneapolis, MN 55455

**Supplemental Materials and Method:**

Naloxone hydrochloride dihydrate was purchased from LGM Pharma (LGM Pharma, FL) and subsequently converted to the corresponding free base (2) via acid−base extraction with saturated aqueous sodium bicarbonate (NaHCO3). 1-[3,5- Bis(trifluoromethyl)phenyl]-3-[(1R,2R)-(−)-2-(dimethylamino) cyclohexyl]thiourea was obtained from Strem Chemicals, Inc. (Newburyport, MA). (3S)-cis-3,6-Dimethyl-1,4-dioxane-2,5-dione (L- lactide), anhydrous dichloromethane (CH2Cl2), dichloroethane (DCE), and toluene (PhCH3) were purchased from Sigma-Aldrich (St. Louis, MO). Naloxone was dissolved in 0.9% saline at a dose of 10 mg/kg. Morphine sulfate, purchased from Sigma-Aldrich (St. Louis, MO), was dissolved in 0.9% saline at a dose of 10 mg/kg. Doses were determined based on previous publications.22 Water was purified via a Millipore Synergy water purification system. All reagents and solvents were used as received unless otherwise noted. 1 H NMR spectra were measured in deuterochloroform (CDCl3) or DMSO-d6 on a Bruker Avance 500 MHz spectrometer. Chemical shifts are reported in ppm employing the residual solvent resonance as the internal standard (CHCl3 δ 7.26 ppm, DMSO δ 2.50 ppm). UV−vis spectra were measured on a DeNovix DS-11 spectrophotometer using a 10 mm quartz cuvette. Gel permeation chromatography (GPC) was performed using a Waters GPC system equipped with a Waters 2410 refractive index detector. A Waters pump and a Styragel HR 3 column (7.8 × 300 mm) were used with THF as the mobile phase solvent. Separations were carried out at 35 °C with a flow rate of 1.0 mL/min. Polystyrene standards (Mn = 500−300 000 Da) were used for GPC system calibration. LC−MS analysis was performed on a Dionex Ultimate 3000 uHPLC system coupled to a Thermo Scientific TSQQuantum Access MAX triple quadrupole mass spectrometer. Reverse-phase chromatographic separation was accomplished on an Agilent ZORBAX Eclipse Plus C18 column (3.5 μm, 100 mm × 4.6 mm) with acetonitrile (CH3CN) and water (H2O), modified with 0.1% formic acid, as the mobile phase solvents. The standard HPLC method consisted of a linear gradient from 1 to 95% CH3CN over 5 min followed by a hold at 95% CH3CN for 1 min and then a re- equilibration at 1% CH3CN for 2.5 min. (total run time = 10 min, flow rate = 0.400 mL/min, injection volume = 10 μL, Tr naloxone = ∼5.2 min).

**Synthesis of NLX-PL(G)A:**

The preparation of our covalently loaded naloxone nanoparticles consists of a two-step process involving the synthesis of a naloxone-containing polymer and the subsequent precipitation of that polymer to form well-defined nanoparticles. To arrive at the targeted formulation for in vivo evaluation, three polymers of varying cyclic aliphatic ester monomer composition (i.e., rac-lactide and glycolide) were first synthesized according to our previously described solvent-free organocatalyzed ring opening polymerization (ROP) protocol as illustrated in Scheme 1. The desired ratio of monomers was pre-melted at 130 °C under inert atmosphere followed by the addition of a mixture of the naloxone initiator and thiourea organocatalyst **X**. After the prescribed time, the crude reaction was cooled to ambient temperature, dissolved in CH_2_Cl_2_ and the polymers were precipitated into cold isopropyl alcohol. The resulting precipitate was dried under reduced pressure and then subjected to flash chromatography to remove any residual naloxone, thus affording the purified polymer products in moderate yield.

Gel permeation chromatography (GPC) analysis showed good control over molecular weight and dispersity in the two polymer variants (Figure S1 and Table 2). Interestingly, incorporation of glycolide into the polymer backbone appears to result in an increase in both the molecular weight and molecular weight distribution in the examples being investigated. The structure and degree of polymerization (DP) for the three polymers were also confirmed via ^1^H NMR analysis. Resonances corresponding to the protons from the various sections of the polymer scaffold are assigned in Figure S2 and color coded for clarity. Polymer DPs were calculated from the ratio of the integrations measured for the methine (***a***) and methylene (***b***) proton resonances of lactide and glycolide monomers, respectively, relative to the vinyl methine peak of the naloxone polymer chain end (***c***). These values, shown in Table 1, were then employed to determine the drug loading of naloxone using standard polymer molecular weight analysis techniques.

**Figure S1.** Gel permeation chromatography traces for NLX-PLA and NLX-PLGA-based polymers


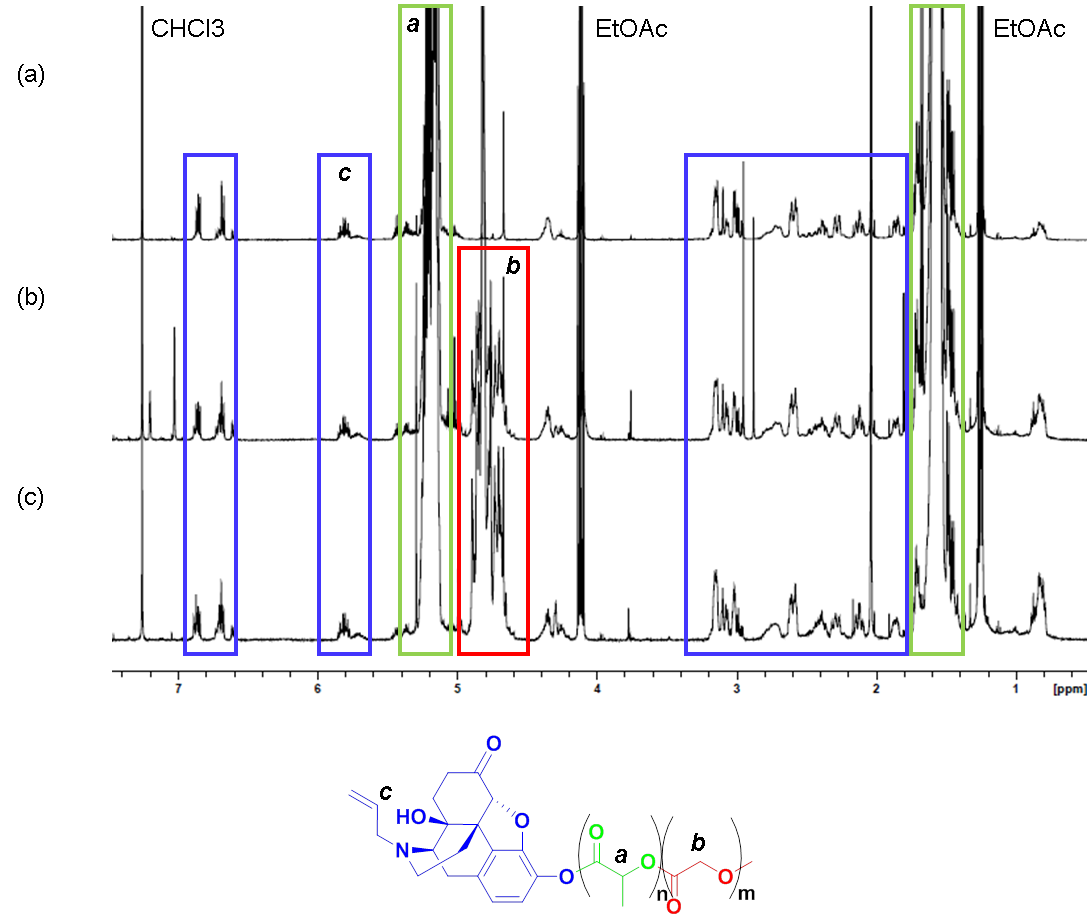


**Figure S2.** ^1^H NMR analysis of polymer products from organocatalyzed ROP of lactide and glycolide in CDCl_3_. (a) NLX-PLA, (b) NLX-PLGA (80:20), (c) NLX-PLGA (60:40)

The resulting purified polymers were then formulated into the desired NLX-NP compounds via a modified nanoprecipitation technique. Polymers were taken up in acetonitrile and added slowly dropwise via syringe pump to a 0.3% aqueous solution of poly(vinyl alcohol) (PVA_MW~6000_) with vigorous stirring. The initial small-scale batches of precipitated nanoparticles were isolated via centrifugation and lyophilization. However, due to poor recoveries on the larger scale preparations needed to supply PK and *in vivo* studies, we turned to dialysis as a method to ensure full NP recovery. Dialysis with 50 kDa MWCO membranes dramatically increased the recovery of NPs.

Naloxone content of the NPs was analyzed via UV-Vis spectrophotometry. To determine NLX-NP concentrations, a standard curve was prepared using a range of NLX from 0.156 – 5 mg/mL. NP samples (5 mg/mL) were then hydrolyzed in 1 M NaOH for 24 h at 50 °C and absorbance values were measured for the various samples. From these values, the corresponding concentrations and thus % loading of each NP sample could be determined. Naloxone loadings for the three NPs are listed in Table S1.

**Table S1.** Naloxone loading in NLX-NP based on UV-Vis analysis.

| **NP** | **NLX loading (%)** |
| --- | --- |
| **NLX-PLA** | **9** |
| **NLX-PLGA (60:40)** | **6** |

Further characterization of the NLX-NP precursors was accomplished through dynamic light scattering (DLS), which measures the size distribution of the particles. As presented in Figure 3 and Table 3, DLS measurements showed a uniform particle size distribution for the three NLX-NP formulations along with a relatively narrow polydispersity index (PDI).

**
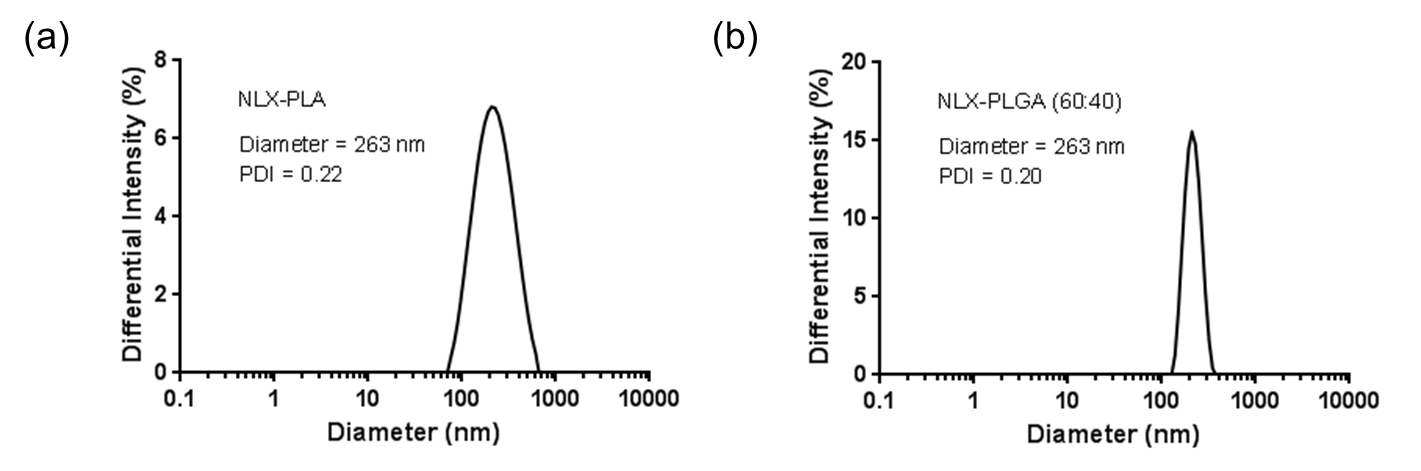
**

**Figure S3.** Dynamic light scattering size distribution analysis of (a) NLX-PLA, (b) NLX-PLGA (60:40) precursor nanoparticles.

**Table S2.** Full DLS Characterization Data for NLX-NP precursors Formulations.

| **Formulation** | **Diameter (nm)*^a^*** | **PDI*^b^*** |
| --- | --- | --- |
| NLX-PLA | 263 | 0.22 |
| NLX-PLGA (60:40) | 263 | 0.20 |

*^a^*Average of three distribution intensity measurements

*^b^*Average of three measurements

**Figure S4:** *Study design of Experiment 2.* Rats were baselined on a hotplate set to 54°C and monitored on oximeter (MouseOX) for oxygen saturation (% SaO_2_ and heart rate) prior to experiment on each day. Then, 0.1 mg/kg SC fentanyl was given at t=0, 6, 24, and 48 hr. Fifteen minutes after fentanyl administration, rats were monitored on the hotplate for antinociception and via oximetry. Immediately afterwards at t=17 min (but not at t=6, 24, or 48 hr), rats received a single IM dose of 10 mg/kg naloxone, nalmefene, or cNLX-NP_100:0_. On Day 1 (t=30 min), rats were monitored on the hotplate and oximeter to demonstrate that all formulations rapidly reversed fentanyl effects. On Day 2 (t=24 hr), rats were tested on the hotplate and oximeter 15 minutes after fentanyl exposure and any rats that had <90% SaO_2_ were given a 0.1 mg/kg SC dose of naloxone to reverse fentanyl-induced respiratory depression. On Day 3 (t=48 hr), rats were tested on the hotplate and oximeter 15 minutes after fentanyl exposure and immediately euthanized to measure fentanyl, naloxone, and nalmefene levels in serum and brain via LCMS.
